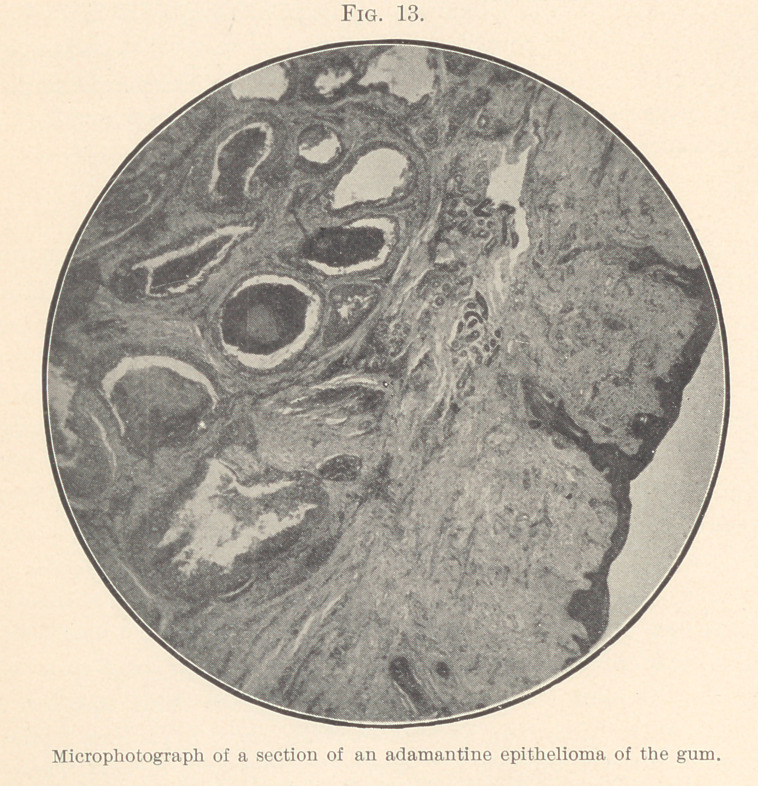# Bone-Cysts: A Consideration of the Benign and Adamantine Dentigerous Cysts of the Jaw and Benign Cysts of the Long Pipe Bones

**Published:** 1904-12

**Authors:** 


### Reviews of Dental Literature.

BONE-CYSTS: A CONSIDERATION OF THE BENIGN AND ADAMANTINE DENTIGEROUS CYSTS OF THE JAW AND BENIGN CYSTS OF THE LONG PIPE BONES.[1] By Joseph C. Bloodgood, Baltimore.

Recent accumulated experience, in the ultimate results after amputation for sarcoma of the long pipe bones, and complete resection for sarcoma of the upper and lower jaw, has demonstrated that local recurrence is unusual, but death from internal metastasis is common in a certain number of cases. When these cases are studied pathologically it is found that the patients who have remained well suffered from special types of sarcoma essentially different from the tumors removed from the patients who ultimately succumbed to internal metastasis. In other words, we were not accomplishing

---

[1] Read at the fifty-fifth annual session of the American Medical Association, in the Section on Pathology and Physiology. We are under obligations to the *Journal of the American Medical Association* for the loan of cuts illustrating this article.

a cure in the latter group of cases even after amputation at the highest joint, because internal metastasis takes place early and is present at the time the patient seeks surgical treatment, and we were subjecting the first group of patients to an unnecessarily extensive operation.

In 1899 [1] I discussed the literature and the experience of the surgical clinic at the Johns Hopkins Hospital in regard to the different relative malignancy in sarcoma of bone, and that in certain varieties much less extensive operations would accomplish a cure with as great a certainty as amputation at the highest joint.

Experience has demonstrated that in some cases curetting is sufficient; for example, the benign bone-cysts of the long pipe bones, dentigerous cysts of the jaw, and medullary giant-cell sarcoma. The latter was advocated many years ago by Koenig. In other cases resection, the extent of which is indicated by the local infiltration of the disease. For example, the various forms of epulides of the upper and lower jaw; the periosteal and medullary giant-cell sarcoma; the periosteal fibrosarcoma and osteosarcoma; the myxochondrosarcoma and a special tumor of the jaw,—the cystic adamantine epithelioma. Such local resections as against amputations have been advocated by von Mikulicz, Weisinger, Morton, Karewski, Hinds.[2]

Amputation is indicated in these varieties of sarcoma only when the necessary resection would result in a useless limb. Infiltration of muscle is not a positive indication for amputation. In this group of cases amputation at the highest joint, except due to the position of the tumor, is never indicated.

In April, 1901, in a discussion before the Philadelphia Academy of Surgery,[3] I advocated this more conservative procedure. Since then the further experience of Dr. Halsted's clinic and my own and the reading of the literature have accumulated additional facts justifying the more conservative operation in this group of sarcoma of bone, of relatively low malignancy.[4]

In December, 1902, I removed with the curette a large medullary giant-cell sarcoma filling and expanding the upper third of the

---

[1] Progressive Medicine, December, 1899, p. 234.
[2] Ibid., pp. 38–42.
[3] Annals of Surgery, 1901, vol. xxxiv. p. 94.
[4] Progressive Medicine, 1902, pp. 151–186.

tibia.[1] This patient has no evidence of recurrence and a limb with unimpaired function.

The experience of the surgical clinic in tumors of the jaw and long pipe bones can be expressed in a few words.

In Group 1 the patients have remained well since operation, the time varying from six months to twelve years. There has been a slight operative mortality, and a few cases in which, on account of the size and position of the tumor, the disease was considered inoperable.

*Benign Cysts of Long Pipe Bones.*—Three cases; one died after operation (Fig. 2); two cases are well.

*Benign Dentigerous Cysts.*—Ten cases (upper jaw, four; lower jaw, four; ethmoid, two cases). Inoperable, no cases. Death after operation, two cases (Figs. 5 and 6). In these two instances a complete resection was performed. Well, eight cases. In these eight cases the operation consisted in partial removal of the bony wall, curetting and packing.

*Adamantine Epithelioma.*—Twelve cases; inoperable, one case (Fig. 8). Death after operation, one case (Fig. 9). Well, ten cases. In one instance there was a second operation for local recurrence; this patient has remained well eight years since the second operation.

PERIOSTEAL SARCOMA.

*Epulis.*—Twenty-three cases; upper jaw, thirteen; lower jaw, ten. One patient died of pneumonia; in this case, an extensive giant-cell tumor of the lower jaw, it was necessary to do tracheotomy and perform a complete resection of the jaw. Well, twenty-two cases. In all of these cases the operation consisted of removal of the tumor with the alveolar border of the jaw. In only one case was there a local recurrence, and this patient has remained well since the second operation.

*Spindle-Cell Fibrosarcoma or Myxosarcoma.*—Eight cases; antrum, three, all well; lower jaw, three, two well, one died of pneumonia after complete resection; orbit and antrum, two cases; one, a young child, died after an extensive operation; the other, also a child, has remained well since the less extensive operation, two years.

*Osteosarcoma.*—(I employ the term osteosarcoma only in those periosteal tumors in which new bone formation predominates.) Eight cases; lower jaw, four, two well, two refused operation; upper jaw, two, both well; long pipe bones (humerus and fibula), two, both well.

*Giant-Cell Sarcoma.*—Those situated on the jaw and called epulis have already been considered. It is a rare tumor of the long pipe bones. We have observed three cases: Ulna, one case, resection, well twelve years. Tibia, upper third, two cases, both well; in both the tumor was recurrent;

[1] Johns Hopkins Hospital Bulletin, May, 1903.

in one the tumor was excised without destroying the continuity of the tibia, in the other the limb was amputated at the thigh.

MEDULLARY SARCOMA.

*Giant-Cell Tumors (Myeloma)*.—Eight cases, all well; lower jaw, one; long pipe bones, seven.

*Myxochondrosarcoma.*—Three cases; one involving the sacrum, inoperable; one involving the upper third of the humerus, well; one of the femur, death two years after operation from tuberculosis of the lungs.

Seventy-eight bone tumors in this group are either benign or of relatively low malignancy. Sixty-nine cases are well, two inoperable; two refused operation; seven died after operation. In six of these cases the tumor involved the upper or lower jaw. In these six cases, I believe, a less extensive operation could have been performed which would have reduced the dangers of the operation, but not the probabilities of an ultimate cure.

Group 2 includes patients who have not been cured, either because the condition was inoperable when they presented themselves at the clinic, or because of death from internal metastasis after operation.

PERIOSTEAL TUMORS.

*Spindle- and Round-Cell Sarcoma.*—Six cases; lower jaw, two; long bones, four.

*Perithelial Angiosarcoma.*—Long bones, two cases.

MEDULLARY TUMORS.

*Spindle- and Round-Cell Sarcoma.*—Long bones, four cases.

*Perithelial Angiosarcoma.*—Long bones, two cases.

In these fourteen cases a complete operation was performed. In the twelve cases of the long pipe bones a high amputation; in the two jaw cases, an extensive resection. In every case death has taken place, usually within a year after the first symptom of the tumor. In only one case was the duration of life longer than two years.

*Sarcoma of the Upper Jaw involving the Antrum.*—Clinical diagnosis: Six cases, all inoperable.

*Carcinoma of Upper Jaw.*—Twenty-one cases; inoperable, sixteen; one death of pneumonia; remainder not cured.

We have, therefore, observed forty-one cases of tumors involving bone of a relatively high malignancy, none of which has been cured, as compared with seventy-eight of a relatively low malignancy, of which sixty-nine are well.

These facts demonstrate the hopelessness of the more malignant varieties of bone tumors.

Surgeons who take the view advocated in this paper must educate themselves to recognize clinically, or through the Röntgen negative, or at the exploratory incision, the different varieties of sarcoma of bone, and govern the extent of the operative procedure by the relative malignancy and extent of the tumor.

In this paper I shall discuss only the benign bone-cysts of the long pipe bones, the dentigerous cysts of the jaw, and the cystic adamantine epithelioma.

BENIGN BONE-CYSTS.

The benign bone-cysts of the long pipe bones are rare tumors. Up to the present time we have observed but three cases in the surgical clinic.

CASE I.—Bone-cyst of the humerus. White girl, aged seven, tumor one year, pathologic fracture. Operation, curetting and drainage, June, 1904, ten months, well.[1] I saw this patient in August, 1903. The parents gave the following history:

*History.*—The apparently healthy child one year ago fractured the upper third of the humerus of the right arm after a slight fall. The fracture was treated by their family physician and united. After the dressing was removed a swelling was observed which has never disappeared. If the swelling had been present before the fracture, it had not attracted the attention of the parents or the physician who treated the fracture. For a year there were absolutely no symptoms except swelling. Three days ago, after a very slight fall, the child refused to use the arm because of pain, and for this reason was brought to the surgical clinic.

*Examination.*—On examination there was a fairly uniform expansion of the upper third of the humerus, easily seen, greatest towards the surgical neck. On palpation the soft parts were normal, but one could feel the normal shaft of the humerus expanding into a thin shell of bone. The surface of this shell of bone was not smooth like the normal shaft, but irregular. In a few places one could elicit definite parchment crepitation, first described by Dupuytren, or what I have called "ping-pong ball" crepitation. The arm was very tender.

Clinically, a diagnosis of bone-cyst was suggested, because so far in my experience every sarcoma of the more malignant type in children at this age had caused death by internal metastasis within a year.

The only medullary tumors of the long pipe bones which, in their growth, expand the bone and produce a definite shell, are the bone-cysts, the myxochondrosarcoma and the giant-cell sarcoma. Of the latter two we had no observations in the clinic, in patients

---

[1] Progressive Medicine, December, 1903, p. 191.

Fig. 5.

Photograph of the alcohol specimen of an excised lower jaw, in which the dentigerous cysts are multiple.

Fig. 6.

This illustrates the huge size which a dentigerous cyst of the upper jaw may reach.

Fig. 7.

Photograph of the alcohol specimen of Fig. 6 after removal.

Fig. 8.

Colored man, aged fifty-four; tumor of twenty years' duration.

at this age, and so far I have been able to find none in the literature. The Röntgen negative is illustrated in Fig. 1.

*Operation.*—At the exploratory operation the tissues were normal until the periosteum was separated. The bone beneath was irregular and varied in thickness from 1 to 4 mm. On removing a piece of the shell of bone a cavity was exposed filled with blood; there was no connective tissue lining, and no evidence of cartilage, but as only sufficient bone was removed to allow curetting of the cavity, one cannot exclude the possibility that cartilage was present in some part of the wall. After curetting, the cavity was partially packed with gauze, the remainder allowed to fill with blood-clot. Eight weeks after operation an X-ray picture demonstrated that the cavity was almost completely filled with new bone.

The origin of these bone-cysts has been demonstrated by Virchow, Zeronie, Schlange, Koenig and others to be due to liquefaction of misplaced islands of epiphyseal cartilage, and in the majority of cases cartilage has been found in some parts of the wall.[1] In the two other cases observed in Dr. Halsted's clinic cartilage was demonstrated in the wall of the cyst.

Case II.—Colored woman, aged thirty-seven. Expansion of the lower end of the right femur, four years.

*History.*—The swelling shown in Fig. 2 reached its greatest height about one year after the onset, and during the last three years there has been little or no increase in size. This patient refused amputation. Five years and eight months later she returned to the clinic.

*Examination.*—The tumor had increased in size, but had not changed its characteristics. The shell of bone was rough, similar to Case I., but thicker, and one could not elicit parchment crepitation. The X-ray showed a shadow somewhat similar to Case I. The diagnosis lay between a bone-cyst and a myxochondroma. The long duration and the preservation of the shell of bone excluded a malignant bone tumor.

*Operation.*—The findings at the operation by Dr. Follis, the resident surgeon, were similar to the case just discussed, except that cartilage was present within the shell of bone in many parts of the wall.

Case III.—The age, clinical history, appearance, and X-ray shadow were almost identical in this patient with Case I., except that the first pathologic fracture had been five years instead of one year before the patient came under observation. The expansion of the upper two-thirds of the femur in this case was produced in the upper portion by cartilage, in the central portion by a cyst filled with blood, and in the lower portion by a fibromyxomatous connective tissue which extended down the medullary cavity of the femur some distance below the point of its expansion (Fig. 3).

---

[1] Progressive Medicine, December, 1899, p. 236.

Dr. Halsted exhibited this patient and the specimen at a recent meeting of the Johns Hopkins Hospital Medical Society. Later a detailed report, with the interesting histologic findings, will appear. The extensive formation of fibromyxomatous connective tissue histologically like ostitis fibrosa, described by von Recklinghausen, is, I think, a unique finding in both cysts.

Koch [1] gives the most complete *résumé* on the subject of bone-cysts, collecting from the literature, in addition to his one observation, twenty-two cases.

Heineke [2] has recently published the first case of multiple bone-cysts in which X-ray negatives were made. He is inclined to the conclusion that the cystic degeneration is part of a general osteomalacia. I have recently learned that Dr. Goldthwaite, in Boston, has under observation, confirmed by X-ray studies, a similar case of multiple bone-cysts, in which there is no doubt clinically as to the presence of osteomalacia.

The etiology of these multiple cysts is apparently entirely different from the single cyst, but in every case numerous X-rays should be taken to exclude multiple cysts. Codman,[3] in Boston, reports a very interesting tumor in the digital phalanx which undoubtedly represents the cartilage stage of the beningn bone-cyst.

The benign cyst of the long pipe bones cannot always be recognized clinically, nor does the X-ray negative differentiate it from medullary giant-cell sarcoma or the myxochondrosarcoma. As a rule, these three tumors can be distinguished from the more malignant and rapidly growing medullary sarcoma. However, if there is any doubt, one should never proceed with an amputation without excluding these tumors of less malignancy by an exploratory incision.

BENIGN DENTIGEROUS CYSTS.

Ten cases. Upper jaw, four cases;. lower jaw, four cases; ethmoid, two cases. The age of the patients varied from six to thirty years; four were under fifteen years of age, six between twenty and thirty. The duration of the tumor varied from three months to thirteen years. Whether the tumor is situated in the

---

[1] Archiv f. klin. Chir., 1902, vol. lxviii. p. 976.

[2] Beiträge z. klin. Chir., 1903, vol. xl. p. 481.

[3] Boston Med. and Surg. Journal, vol. cl., No. 8, p. 211, February 25, 1904.

Fig. 9.

Dentigerous cyst of upper jaw. Death followed complete excision.

Fig. 10.

Photograph of resected lower jaw.

Fig. 11.

Patient from whom tumor shown in Fig. 10 was removed.

upper or lower jaw, it is of slow growth and usually painless. There is a slow expansion of the jaw, and on palpation one can feel a smooth, thin shell of bone. Usually there is parchment crepitation. At the exploratory incision the periosteum is normal. The outer shell of bone is smooth; lining the bone there is a thin, vascular connective tissue membrane. The contents of the cyst is usually a blood-stained serum.

Microscopically, one finds frequently cholesterin crystals, blood-corpuscles, and degenerated cells, which suggest epithelium. Histologically, however, I have never been able to demonstrate an epithelial lining.

Usually the cyst is single; now and then there are thin partitions. In a few cases the cysts are multiple. Complete resection is unnecessary. Partial resection with curetting and drainage will accomplish a cure. In three of our cases a non-erupted tooth was found in a recess of the cyst. These dentigerous cysts are apparently due to the distention of the connective tissue capsule of a non-erupted tooth.

Case IV.—Fig. 4. The patient was a white boy, aged fifteen.

*History.*—The swelling of the body of the lower jaw was of three months' duration. Because of the painless and uniform expansion, the distinct shell of bone, and parchment crepitation, I made the diagnosis of a dentigerous cyst.

*Operation.*—The exploratory incision revealed the pathologic findings already described. The outer expansion was cut away with the chisel without destroying the continuity of the lower jaw. The connective tissue membrane and tooth were removed. The bone cavity was then curetted, allowed to fill with a blood-clot, and the skin incision closed. The wound healed *per primam.*

*Result.*—June, 1904, six years after operation, there is a slight depression in the jaw at the site of the scar, but no other deformity.

Case V.—Fig. 5. The patient was a white girl, eight years of age, the tumor of some years' duration.

*Operation.*—Complete resection was done. The child's hæmoglobin was but fifty-two per cent. at the time of the operation, and although there was no loss of blood the patient died of shock. The tumor was a very large one and extended from the zygoma almost to the symphysis of the jaw. I believe incision and curetting would have been sufficient in this case.

Case VI.—Fig. 6. The patient was a colored girl, nineteen years of age, the tumor of thirteen years' duration. Complete resection was performed (Fig. 7). The patient died at the end of the third week from abscess of the lung.

In the remaining five cases of dentigerous cysts, two of the upper jaw and three of the lower, the operation was similar to that followed in Case IV. The patients recovered and have remained well for from two to eight years since operation.

The two cases of cysts of the ethmoid bone presented themselves clinically with a tumor projecting from the angle between the nose and the supraorbital ridge, producing slight exophthalmos. On palpation the tumor was smooth and presented a thin shell bone giving parchment crepitation. In both, partial excision with curetting and drainage was performed. The patients have remained well, one four years and the other eighteen months since operation.

### ADAMANTINE EPITHELIOMA.

Of these there were twelve cases. In four the tumor projected from the alveolar border of the jaw (three lower, one upper). The tumor was covered with normal mucous membrane and did not invade the bone. In eight cases the epithelial tumor was situated within the body of the jaw (one upper and seven lower), and in its growth produced an irregular expansion very similar to a dentigerous cyst. The age of onset varied from eighteen to sixty-one years. The majority of cases were twenty and thirty-five years of age; the duration of the tumor from seven months to twenty-nine years, the majority from six to twenty years. In one case the condition was considered inoperable (Fig. 8). In the remaining eleven cases a complete resection of the diseased area was made. Nine cases have remained well for from one to twelve years. In one instance there was a local recurrence, but this patient has remained well eight years since the second operation. One patient (Fig. 9) died after complete excision of a huge tumor involving both upper jaws.

This epithelial tumor is apparently of a very low grade of malignancy. In none of our cases was there metastasis to the glands of the neck. The tumor can be differentiated from the more malignant neoplasms of the jaw by its very slow growth. The adamantine epithelioma involving the alveolar border cannot always be differentiated clinically from the connective tissue epulis. The epulis is more apt to be associated with ulceration of the mucous membrane.

When the adamantine epithelioma originates in the body of the jaw and produces expansion, with the formation of a thin shell of

Fig. 12.

Microphotograph of a section of an adamantine epithelioma, involving chiefly the alveolar border of the lower jaw. In this section the basal columnar cell and its various morphologic changes towards the centre of the alveolus are well shown. (Sent me by Dr. Steensland.)

Fig. 13.

Microphotograph of a section of an adamantine epithelioma of the gum.

bone, it cannot be differentiated from a benign dentigerous cyst until the exploratory incision is made. Then, when the shell of bone is incised, we do not find a cavity, but a white, finely granular tumor containing connective tissue trabeculæ, and usually many small and large cystic cavities. The gross appearance is well illustrated in Fig. 10. This patient (Fig. 11) was a colored man, forty-two years of age, tumor of eight years' duration. The patient has remained well three years since operation.

The microscopic appearance is well illustrated in Fig. 12.

Case VII.—Fig. 13. The patient was a white woman, aged fifty-two.

*History.*—Four years ago, when forty-eight years of age, she observed a tumor like a gumboil on the outer side of the alveolar border of the left lower jaw, opposite the canine and first molar teeth. At the end of two years, when it had reached the size of a hickory-nut, it was removed. A local recurrence took place within six months.

*Examination.*—The recurrent tumor is the size of an egg and involves the alveolar border of the left lower jaw from symphysis to within one centimetre of the angle. The tumor is present on both sides of the alveolar border. It is distinctly circumscribed. The mucous membrane at one point has ulcerated, exposing a small cyst.

*Operation.*—The tumor was removed by turning back flaps of mucous membrane which were not adherent to the tumor. Then the tumor and the alveolar border of the jaw were removed in one piece. The patient has remained well since the operation, a period of ten years.

*Appearance of Tumor.*—The fresh appearance was quite typical: white, friable, granular alveoli of various sizes and cysts in a definite fibrous stroma.

Under the microscope (see Fig. 13) one sees the normal mucous membrane of the gum, then a zone of connective tissue, beneath which is the circumscribed tumor. The tumor is composed of branching epithelial alveoli in a connective tissue stroma. Some of the alveoli are cysts lined by the typical basal adamantine epithelium. Other alveoli are solid, with cells showing the various morphologic changes of the adamantine epithelium.

### DISCUSSION.

*Dr. F. J. Hall*, Kansas City, Mo.—It would be of interest to know what distinction, if any, Dr. Bloodgood makes between the cystic bone tumors of the long bones; in particular, two varieties of pathologic bone condition: one is the myeloma and the other is the traumatic myositis ossificans. Several cases of both these conditions have occurred in my own territory, and three of the cases of myeloma—so-called central sarcoma of the bone—have been operated on, and none has recurred. Three cases also of myositis ossificans have occurred wherein the central portions of the tumors were occupied by these cysts, and I should like to know if Dr. Bloodgood has had any experience with these other two varieties of conditions, and what relation they bear to the conditions which he has described.

*Dr. Joseph C. Bloodgood*, Baltimore.—I am inclined to think that what you mean by myeloma is a giant-cell tumor.

*Dr. Hall.*—Yes, sir.

*Dr. Joseph C. Bloodgood.*—Dr. Hall, in his question in regard to myeloma, undoubtedly means the medullary giant-cell sarcoma. This tumor is one of a relatively low grade of malignancy. We have observed about ten cases; all have remained well since operation. I have reported them in the Johns Hopkins Hospital Bulletin for May, 1903. In regard to the question as to the origin of the blood-cysts in the condition called myositis ossificans, they are probably due to hemorrhage. Recently there has appeared in the literature a number of interesting articles and reports of cases on this subject. These I have reviewed in *Progressive Medicine* for December, 1903. With or without a history of trauma an indurated mass associated with some pain and tenderness is observed by the patient in one of the large muscles, most frequently the thigh. The tumor rapidly becomes bony in hardness. In some cases the X-ray demonstrates a zone of normal tissue between the shaft of the bone and the osteoid tissue in the muscle. In other cases the two bony shadows are in contact. This has given rise to two views as to the etiology of the new bone formation in the connective tissue between the muscle bundles. A number of authorities conclude that the bone is a product of detached pieces of periosteum, others that it rises from the connective tissue cells between the muscle bundles. Clinically the condition is not difficult to recognize, especially with the aid of the X-ray. However, when the new shadow rests directly on the shaft of the neighboring bone it will be difficult to differentiate the ossifying myositis from a condition called traumatic exostosis or ossifying periostitis. Blood-cysts have been observed only in the ossifying myositis. Extensive operations are not necessary. One should remove as much of the new bone production as possible without destroying function. Slight local recurrences of the bone formation are to be expected. Fortunately the amount is never great, gives little or no discomfort, and second operations are rarely necessary. I do not think there is any relation between the hemorrhagic cysts observed in myositis ossificans and the cysts of the long pipe bones.

As to the question of the origin of the giant cells, recent investigation would indicate that the periosteal or medullary giant-cell tumor is probably an angioma or an angiosarcoma, and that the giant cells are due to budding of the endothelial cells of the vessels in these very vascular tumors. The most interesting publication on this subject is by Friedlänger.[1] Further investigation, however, should be made of this most interesting tumor.—*Journal American Medical Association.*

[1] Archiv f. klin. Chir., vol. lxvii. p. 202.

## Figures and Tables

**Fig. 1. f1:**
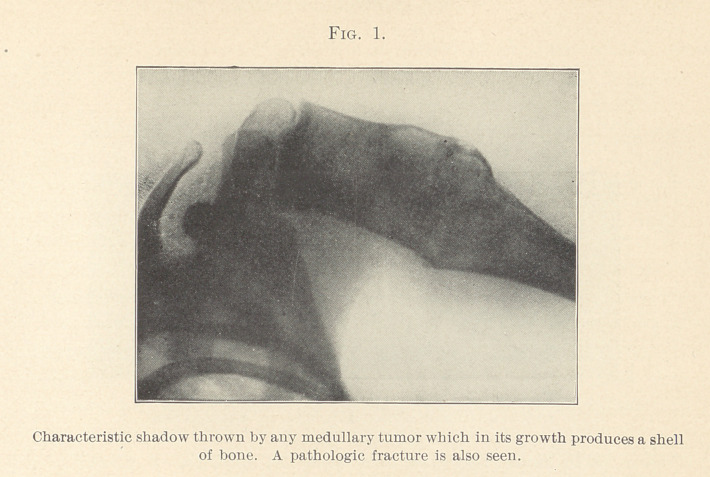


**Fig. 2. f2:**
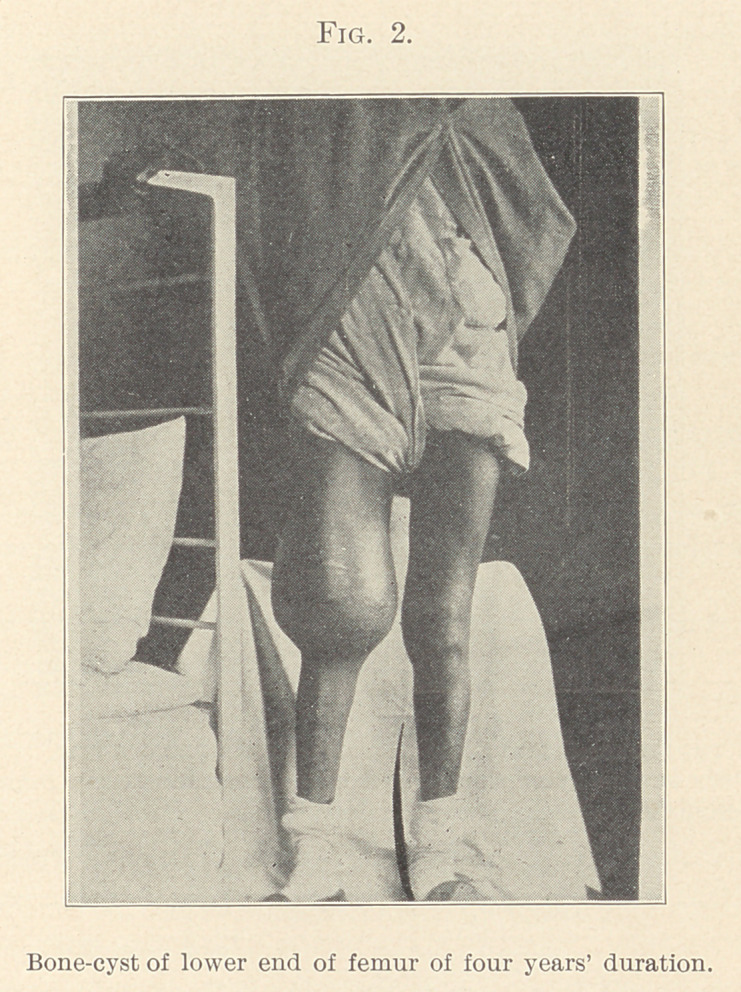


**Fig. 3. f3:**
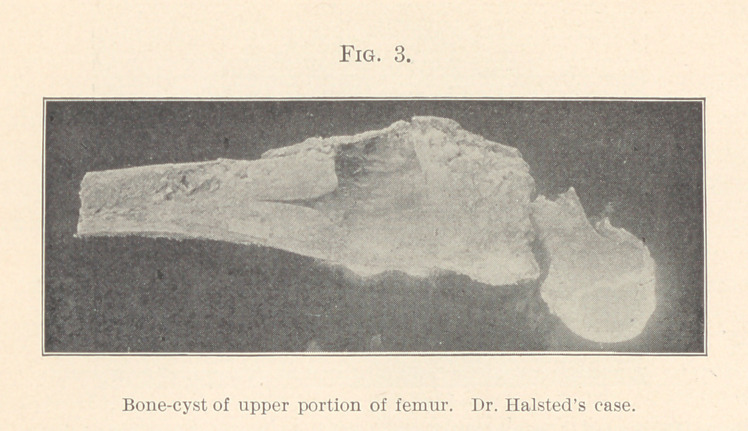


**Fig. 4. f4:**
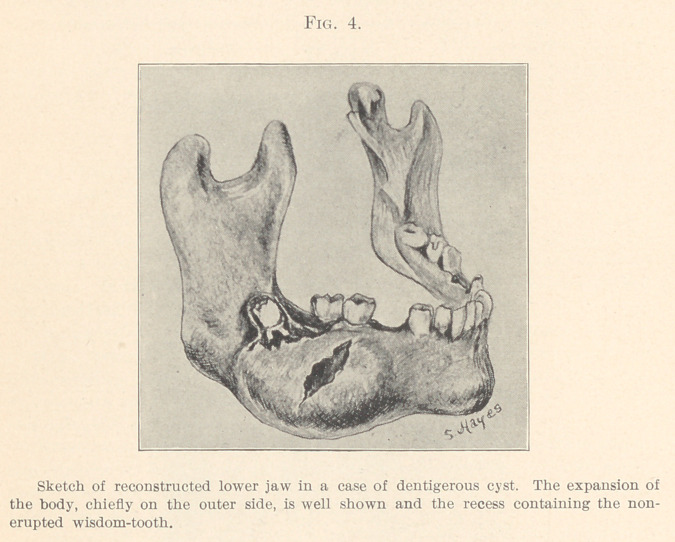


**Fig. 5. f5:**
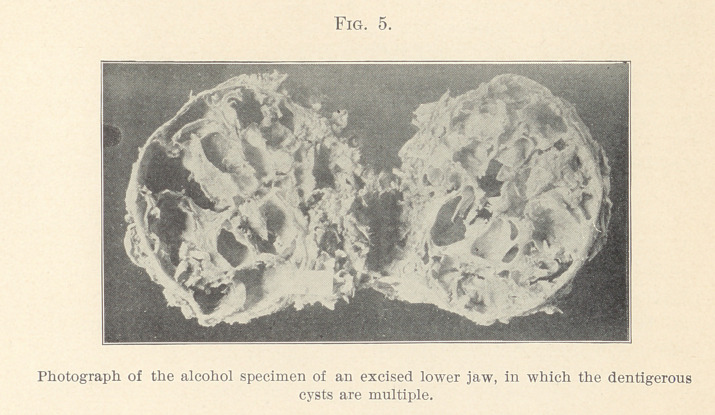


**Fig. 6. f6:**
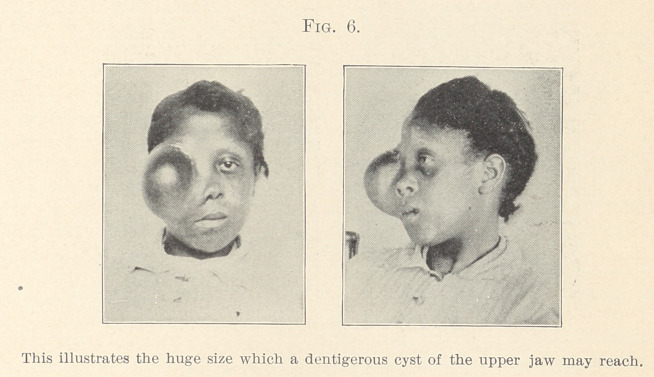


**Fig. 7. f7:**
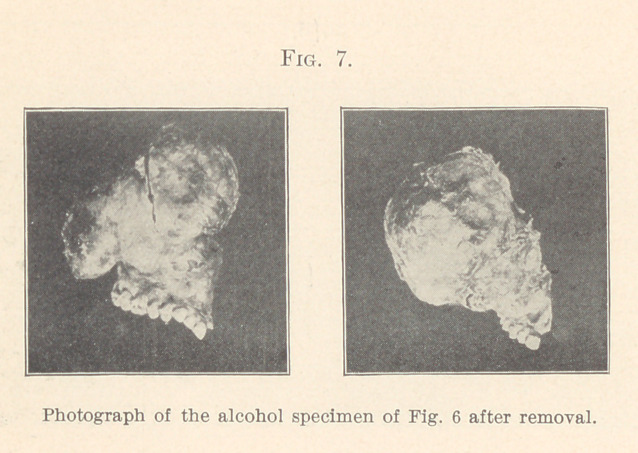


**Fig. 8. f8:**
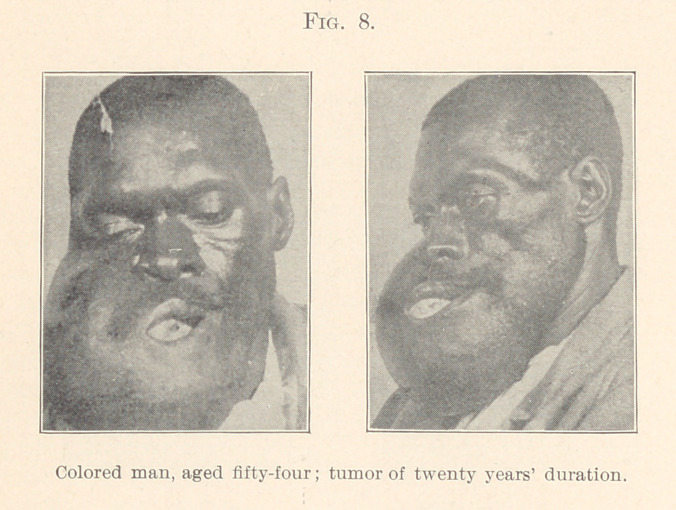


**Fig. 9. f9:**
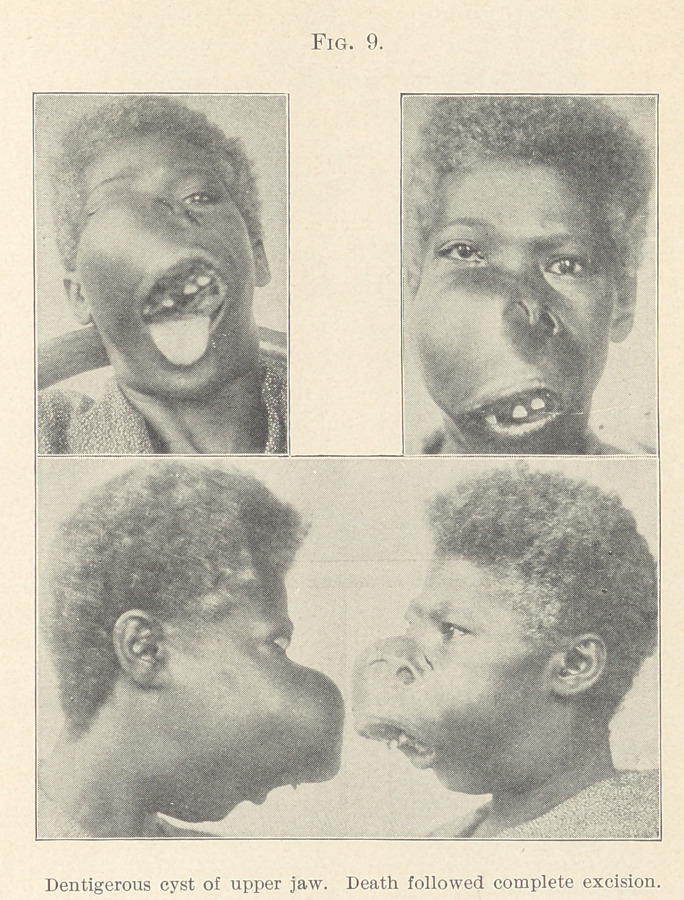


**Fig. 10. f10:**
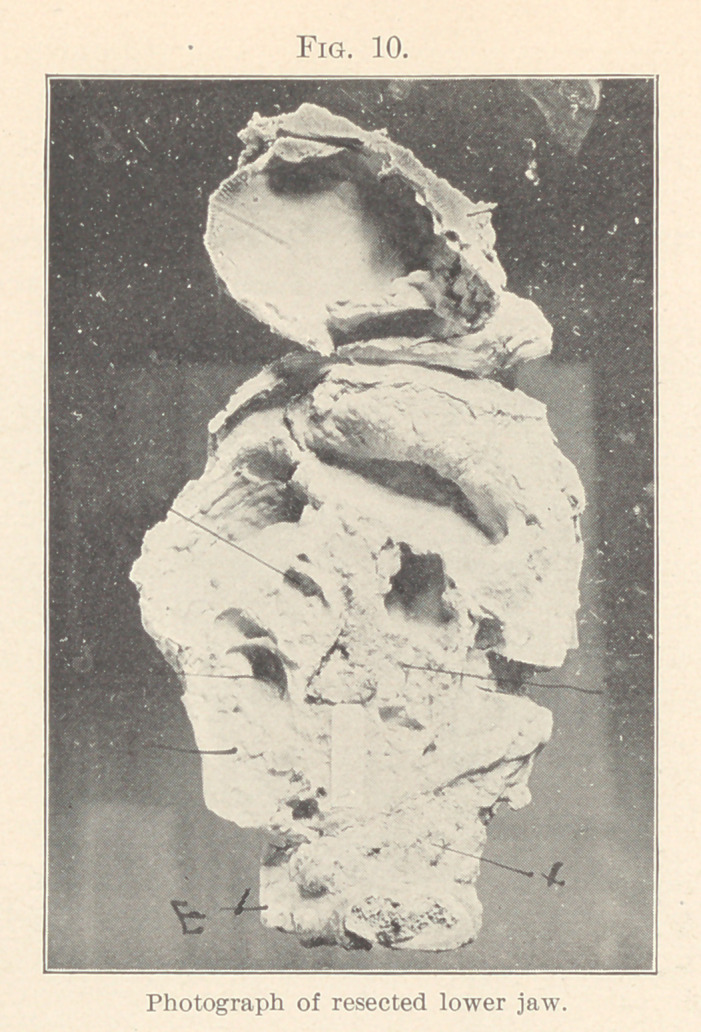


**Fig. 11. f11:**
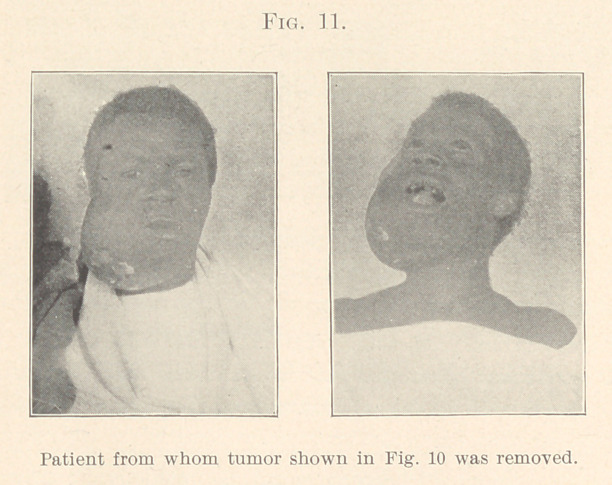


**Fig. 12. f12:**
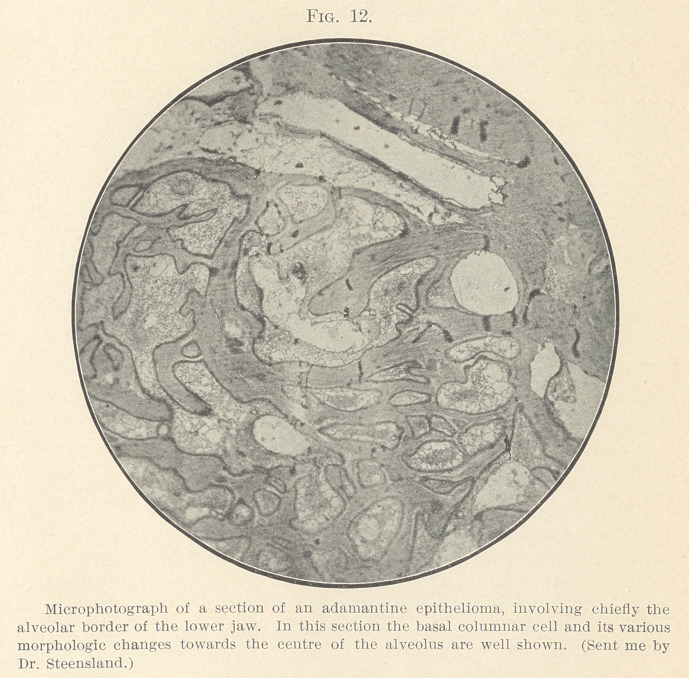


**Fig. 13. f13:**